# Residents’ Spatial Preference for Urban Forest Park Route during Physical Activities

**DOI:** 10.3390/ijerph182211756

**Published:** 2021-11-09

**Authors:** Mengmeng Cai, Chuyun Cui, Lin Lin, Shuyi Di, Zheng Zhao, Yanbin Wang

**Affiliations:** 1 Shanghai Institute of Tourism, Shanghai 201418, China; guxierjiu@163.com; 2 School of Economics and Management, Beijing Forestry University, Beijing 100083, China; cuichuyun@bjfu.edu.cn (C.C.); linlin2001@bjfu.edu.cn (L.L.); 3 College of Tourism, Shanghai Normal University, Shanghai 200234, China; dshy50363@163.com; 4 Economic Development Research Center, National Forestry and Grassland Administration, Beijing 100714, China

**Keywords:** urban forest park, spatial preference, environmental preference, cognitive map, China

## Abstract

Urban parks positively affect the life quality and health of urban residents as well as the environment where they live. When it comes to the design of a future urban forest park, it is necessary to consider the protection of ecological environment, landscape sustainability and practicability. This study explored residents’ spatial preference for urban forest parks based on preference survey data. According to the rating scores obtained for four urban forest park routes during physical activities, this study used cognitive maps and multinomial logit models to figure out the potential influencing factors affecting residents’ spatial preference while they engage in physical activities. The results suggest that forest routes are still the primary choice for urban residents. Although familiarity with the spatial image preference for urban forest parks varied from person to person, residents’ choice of route shows certain commonalities, which was reflected in the sequential cognitive maps obtained from them. In addition, residents’ route preference is influenced by their exercise habits, environmental preference and residential location. There is also a certain correlation between residents’ preference and their characteristics. This study provides additional information for planners, developers, engineers, architects and foresters in building a more suitable environment that is aesthetically appealing and ecologically sound for physical exercising.

## 1. Introduction

Urban forest parks are important leisure areas for urban residents. They are generally used for physical and recreational activities. Studies have confirmed that these functions can positively affect residents’ mood, cognition, behavior and physical health [1]. From the perspective of functions and service types, parks can improve the ecological environment, meet residents’ living needs, improve their physical health and reflect the relationship and interaction between human and nature [2,3,4,5,6]. Besides, urban forest parks also undertake certain social service functions [7]. The Millennium Ecosystem Assessment report proposed by the World Health Organization, the United Nations Environment Programme, the World Bank and other institutions and organizations classifies ecosystem service functions into four aspects: supply, regulation, culture and support, including a large number of descriptions of ecosystems used to provide socialized services. This provides an important basis for establishing the relationship between urban forest parks and residents’ needs.

As far as urban forest parks are concerned, people are more likely to engage in the form of physical or recreational activities when embrace nature. In this process, people’s behavior pattern in the environment is based on their cognition and understanding of the things around them, and it has been proven that participating in physical activities or exercise will be beneficial to people’s physical and mental health as well as their cognitive function. While engaging in physical activities, residents develop a deep or shallow preference for the images of things around their exercise route, which refers to people’s image cognition of the material environment [8,9]. This process involves the reconstruction of the image of the surrounding objects in the brain, which forms a two-dimensional or three-dimensional image of the things or scenes that have happened, and the image of the specific material environment is embodied in a hand-painted cognitive map. As the image preference for respondents reflects their behavior, cognitive maps can generally reflect the main characteristics of the environment from the perspective of residents’ feedback [10]. Therefore, the activities of urban forest park users can be assessed through studying the image preference of urban residents, which has a certain reference value for the design of internal lines, spaces and facilities of urban forest parks.

At present, residents’ preference for urban forest parks has expanded from traditional aesthetic and practical functions to deeper levels, such as pursing psychological and emotional satisfaction. Some studies believe that the simplified mathematical and statistical models constructed from the characteristics of residents and urban forest parks cannot effectively elucidate the complexity of real urban forest parks and the subjective initiative of residents. Since the subjectivity and objectivity of urban forest parks are equally important and the research object has been extended to the “subjective world” of the urban forest park, such as “perceptual space” and “image maps” [11], we believe that empirical analysis has limitations and does not meet the current research needs. Based on cognitive maps and spatial cognitive theory, the cognitive map method has been widely used in decision-making process associated with spatial behavior, thus expanding the research scope of the subjective will of micro-subjects. To some extent, it solves the above problems [12,13,14]. The concept of the cognitive map was first proposed by Tolman (1948) in “The Rat Maze Walking” experiment; this concept was later used in China in the 1980s [15]. Gao (1992) laid the foundation for studying cognitive maps and spatial cognitive theory in China [16]. Since then, research related to cognitive maps has expanded. Some studies have combined the cognitive map method with the questionnaire survey method [17,18,19]. Kevin Lynch, a famous American urban designer, first integrated the concepts of environmental image, public image, readability and imageability into the analysis of urban images. Combining people’s environmental psychology, he used cognitive maps and environmental images to analyze the urban spatial forms of Boston, Los Angeles, and Jersey City. Empirical studies based on it led to the establishment of five image elements of cognitive maps: landmarks, roads, nodes, regions and boundaries. Ever since, numerous studies have conducted extensive theoretical explorations and developed practical improvements to this concept. The urban image research method framed by Lynch was particularly effective for analyzing local areas [20,21,22]. In general, Lynch (1960) provided a comprehensive set of research methods for urban image space, including sampling interviews, drawing cognitive maps and testing the environmental images formed by trained observers. These concepts and methods provide the basis on which our study is developed.

Similar to existing studies, this study posits that understanding public preference will help avoid misleading preference [23]. Extensive researches have been conducted on individual urban forest park preference, especially relating to the influential factors of individual preference. Due to different individual characteristics, family characteristics, degree of specialization and other factors, individual preference varies from person to person [24,25,26,27]. However, investigating it is rather difficult because although multiple attributes cannot exist and play a role independently, their degree of influence has a cross-influence with other attributes [28,29]. Additionally, residents with different personalities have different preference regarding the landscape and structure of urban forest parks. A scientific and reasonable form of urban forest parks should be designed to provide psychological and physical benefits to all types of residents [30,31]. To design a scientific and reasonable urban forest park, planners and architects often face trade-offs between various elements and styles in their work, considering the individual’s different preference for various urban forest park landscapes. In addition, some studies have raised concerns regarding the differences in the preference of residents owing to different temporal and spatial conditions [32,33]. Overall, the public’s preference is rooted in its social status and its own economic, cultural and social capital [34,35]. Therefore, studies on preference of residents for urban forest parks must be combined with a detailed assessment of the interactions between their characteristics and qualities as well as variations of landscapes. On this point, previous studies focused on the structure, design and layout. However, most of them only zero in on the comparison and impact of natural and artificial environments, and they are descriptive discussions that lack quantitative results and objective measurement standards. Studies have suggested that merely separating the natural environment from an artificial one is unlikely to provide an in-depth insight into benefits of the environment for human well-being [36]. Therefore, we should reasonably construct and highlight the naturalness of urban forest park design and ensure that the city’s social and economic functions are not ignored because of the excessive increase in vegetation cove [37].

Changes in preference of residents affect the planning and decision-making processes related to urban forest parks [38]. Therefore, it is of great academic and practical significance to understand people’s preference and factors influencing the utility of urban forest parks. In this respect, no previous study has comprehensively addressed the relationship between individual attributes and urban forest park variations with residents’ preference and choices. This study focuses on the choice of activity routes of urban forest parks selected by residents, and aims to address the following questions: Which urban forest park route environment or combination of preferences do residents prefer, and what are the types and degrees of residents’ preferences? As we mentioned, this study analyzed the urban forest park environment based on four major routes, which residents used while engaging in physical activities.

From the perspective of public cognition and choice, this study considered the Olympic Forest Park in Beijing as the research area. Further, we used cognitive maps and multivariate logit models to study the spatial image preference and preference characteristics of urban forest parks in different locations and for different individual characteristics. The data for this study were obtained from a field questionnaire survey and field interviews. The primary objective of this study was to understand residents’ preference for urban forest parks, especially to explore their preference for typical urban forest park routes when they engage in sports activities, as well as potential influencing factors. In this way, we can emphasize the importance of urban forest park in residents’ life, explore the reasonable form of urban forest park, provide planners, developers and government staff with more useful information about its design and lay the foundation for building a more reasonable and humanized park.

## 2. Research Design

### 2.1. Study Area

**Figure 1:**
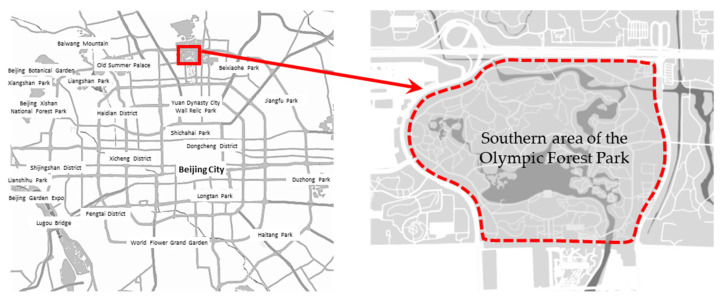
Geographical location of the Olympic Forest Park, which is the study area.

As this study aimed at improving the understanding of public preference for urban forest parks, the Olympic Forest Park, a typical urban forest park, located in an urban area of Beijing was selected as the research site. Olympic Forest Park is located near the North Fifth Ring Road in Chaoyang District, Beijing, and covers an area of 680 hectares, including 380 ha in South Park and 300 ha in North Park. The Olympic Forest Park was built for the opening of the 2008 Beijing Olympic Games. At present, it is a national scenic spot and an important place for leisure, entertainment and exercise for Beijing residents. Olympic Forest Park is rich in forest resources, and boast a greening coverage rate of 95.61%, especially in South Park. Considering accessibility and the number of users, the South Park of Olympic Forest Park was selected as the primary research area (Figure 1).

### 2.2. Survey Location

**Figure 2:**
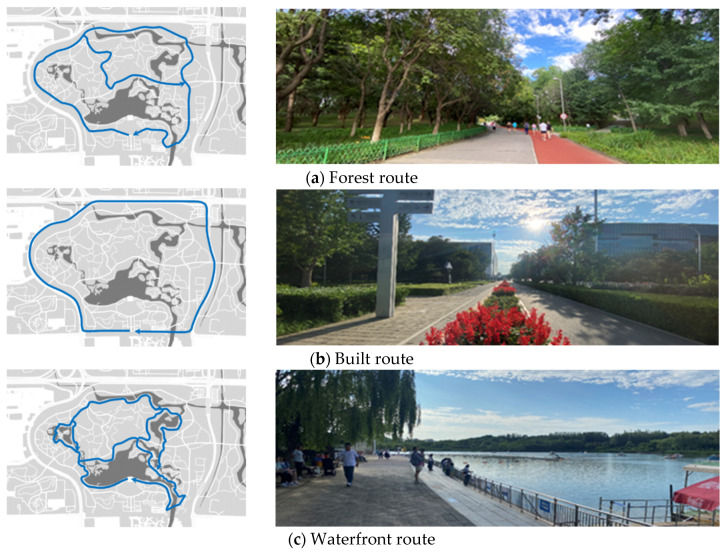

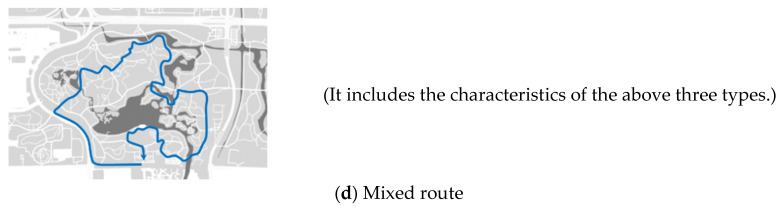
Four routes summarized for residents while they engage in physical activities in the Olympic Forest Park.

As mentioned in this study, a reasonable urban forest park design is often a trade-off between natural and artificial environments to achieve optimal results. Hence, we summarized four routes within the urban forest park to understand the trade-offs between residents’ preference of natural and built routes and to explore the needs of the residents concerning natural and artificial areas (Figure 2). It should be pointed out that this study does not emphasize the architectural principle of urban forest park route, but the atmosphere it creates, the characteristics of different types of routes and the different feelings it brings to residents so as to reflect different residents’ preference.

Of course, residents do not strictly follow the established route given in this study. What this study gives is only the most widely used route of residents. In other words, our definition of route comes from empirical investigation and interview conclusions, and of course, it is also based on the actual use of residents. We hope that we can refine conclusions and summarize laws through these more frequently used and typical routes.

1. The forest route is located in the inner area of the urban forest park, and offers high vegetation coverage, pleasant scenery and fresh air. The forest route is widely distributed and it is suitable for long-distance sports and recreational activities. However, the terrain of the forest route fluctuates and there are many curves along the route, so it is not suitable for night activities. Meanwhile, if the roads become narrow and many people gather together, users will be easily disturbed by others during physical activities while in the forest route. In addition, forest routes are mostly paths and closed routes inside the park. Therefore, residents cannot exit at any time during the exercise [39,40,41].
2. The built route is situated outside the park, and primarily consists of sidewalks on both sides of the municipal roads. The built route is a circular road that forms the boundary of the urban forest park. The route offers a good field of vision during physical activities. At the same time, the road surface of the built route is relatively flat, which makes it conducive to all types of sports, such as running. Unlike the forest route, the built route has no restrictions on entry and exit. There are bus stops, subway stations, shops and other facilities on the roadside, which can meet the various needs of residents. However, the disadvantages of the built route lie in its relatively low vegetation coverage, monotonous landscape and residents’ direct exposure to the sun during exercise, resulting in great sunscreen pressure, especially when the body temperature in summer is relatively high. Besides, the built route is close to motorways, exposing the users to turbid air, loud noise, vehicle exhaust and smoke during exercise. Similarly, the safety of exercising in built route is relatively low due to high vehicle speed on the motorway [42,43].
3. The waterfront route is usually a short route around the lake and is characterized by rich elements (water environment and vegetation) on both sides of the road. Along this route, there are many entertainment facilities, such as cruise ships, boating and other entertainment facilities at the lakeside wharf, which enhance the ornamental and entertainment value of the waterfront route. However, similar to the forest route, this route undulates and thus has many curves, so it is easy to cause wrestling, sprains and other problems when residents perform exercises such as running [44].
4. The mixed route incorporates the characteristics of the above three types of routes. Residents who exercise in this route do not have clear preferences or motives. The mixed route offers a wide range of space, flexible utilization methods and diverse landscapes, rendering it suitable for all people. Residents who exercise in the mixed route can experience different feelings when exercising and can also choose to enter or exit the park at any time, making exercise in this route rather universal and convenient. However, the mixed route also has the disadvantages of the three route types discussed above.

### 2.3. Experimental Procedures

In this study, we adopted a combination of questionnaire surveys and cognitive maps supplemented by interviews. First, we collected relevant data using a questionnaire; before that, the research group designed a unified questionnaire and survey scheme. A preliminary draft of the questionnaire was prepared based on existing literature, expert interviews, and group discussions. Further, cognitive map analysis was performed to evaluate the spatial preferences of residents using the Beijing urban forest park, and a multinomial logit model was applied to analyze residents’ preferences for four urban forest park routes. Hence, we applied this method to analysis the urban forest park in Beijing. Hand-drawn cognitive maps are considered to be one of the direct graphical methods, in which respondents are requested to draw the shape, location and structure layout of various objects [45]. As residents have different emotional memories and subjective impressions of urban forest parks, the image preferences of residents can embody the objective image of urban forest parks. We applied image theory to the study of urban forest parks. Based on this concept, the residents were asked to draw their own cognitive maps of the Olympic Forest Park with the most familiar elements in their impressions and to mark words that they thought were necessary or important to describe the route and their experience. The key elements reflected by residents on their hand-painted cognitive map are usually the most familiar or commonly used parts. As mentioned in this study, important information related to residents’ preferences can be obtained through the extraction and integration of these elements. This study integrated the frequency of different elements in residents’ image preference based on statistical analysis, marked the main elements on a complete map, and then drew sketches reflecting residents’ preferences of urban forest parks.

Owing to difficulties in obtaining information from hand-drawn cognitive maps, we divided residents into two types: (1) residents with a deep preference for the spatial image of the park, who were requested to draw a hand-drawn cognitive map; and (2) residents with a vague preference for the spatial image of the park, who completed the hand-drawn map and were asked to select the most familiar elements on the map. The purpose of these two surveys was to obtain information on how familiar the spatial image preferences of residents were for different types of urban forest park routes. Frequency distribution statistics and comparative analyses were conducted based on the information obtained.

On the other hand, this study used multivariate logit models to study the preference characteristics of urban forest parks in different locations conditions and for different individual characteristics. The multinomial logit model design serves two purposes: to compare the relative importance of different types of routes, and to compare the subjective spatial preferences of residents. In the setting of dependent variables, we take residents’ urban forest park route preferences as options. Based on this, we construct a disordered multivariate logit selection model for calculation, and compare the disordered selection of various types of urban forest park routes of individual residents; and in terms of independent variables, we collected information including gender, age, education level, location of residence, family size and frequency of visits, hoping to further analyze the influencing factors of residents’ choice through multiple logit regression method. Specifically, residents’ heterogeneity in personal characteristics and prior experience, such as gender, age, education level and other factors, will affect their preferences; at the same time, residents will have different preferences for different park forms in different time and space. For example, considering the openness and occlusion of information, the degree of citizenization and familiarity with parks will have an impact on residents’ preferences [46].

The variables and rating scores are shown in Table 1. Based on this, we can reveal the relationship between residents’ individual characteristics and their urban forest park route preference.

As mentioned above, the questionnaire survey environment of this study is located in the Beijing Olympic Forest Park, and four exercise routes were extracted based on the actual situation of residents’ activities. The survey in this study was carried out on each established route. Among them, the road widths of the four routes were not significantly different. Based on selected research areas, questionnaires and corresponding survey methods, field surveys were conducted in September 2021 from 24 September to 28 September, the investigation starts from the opening of the park at 10 a.m. every day, and the end time is determined in combination with the real-time passenger flow in the park on different days. Based on this arrangement, data from Olympic Forest Park were collected. The investigators were all graduate students with professional learning backgrounds and experience in field investigations. Investigators underwent systematic training on research methods and data collection before conducting the survey. The core principles of this study are efficiency and comprehensiveness. These principles made the questionnaire as concise and clear as possible to improve residents’ responsiveness as well as the utility and accuracy of the obtained data. Considering the complex characteristics of Shanghai residents, we select the sample residents according to the combination of stratified sampling and random sampling, so as to improve the representativeness of the sample and reduce the sampling error. At the same time, we take a face-to-face approach in the specific survey links, supplemented by the investigators of the research group to properly explain the possible questions in the questionnaire. After the survey, the research group cross checked the questionnaire three times to ensure the quality of the survey data.

**Figure 3:**
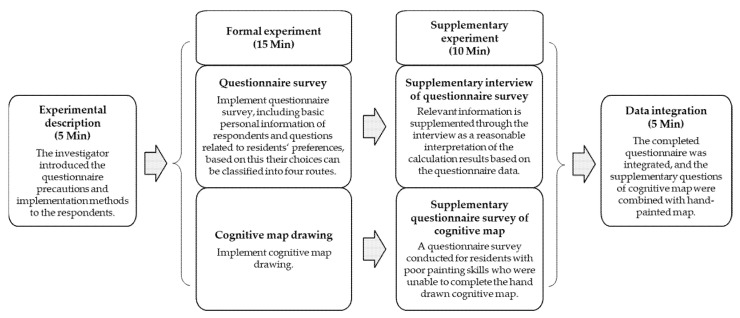
Timeline of the experimental session.

After the survey, there are two main aspects for data sorting: one is to input the conventional paper questionnaire data into the computer and convert it into electronic data; the other is to manually identify the subjective data such as cognitive map and convert and input it into electronic data. These two tasks can be completed quickly with sufficient staff, including data verification and inspection. Overall, 874 questionnaires were collected, of which 803 questionnaires were validated, and 309 effective cognitive map sketches were obtained. The overall effective response rate was 91.88%. It should be noted that we adhere to the principle of random sampling when selecting the interviewed residents. Some residents refused the interview for personal reasons, such as managing children, dating, exercising and not wanting to be disturbed. There is no substantial or significant difference between non respondents and respondents, nor does it affect our random sampling process. The above empirical procedures are shown in Figure 3.

In order to reduce the survey error, we have taken the following measures: first, combined with the research theme, we have correctly formulated the survey plan, scientifically designed the questionnaire, reasonably selected the survey methods, made our survey meet the actual situation of the respondents, and enabled the investigators to clearly implement the established plan.

### 2.4. Data Analysis Method

In this study, Y represents the routes of urban forest park. Y1 to Y4 are the four types of urban forest park routes, which is the basis for residents’ choice. Starting from this, we give two main reasons for using the disordered multiple-choice model for calculation: First, although the routes of urban forest park themselves as dependent variables are orderly, the four types of urban forest park routes are disorderly. For residents, which type of urban forest park route they choose is also disorderly; Secondly, the functions of the selection models used to calculate such problems are similar, but the multivariate probit model is more complex. Therefore, the multivariate logit model with multiple choices is used for calculation.

Multiple logit regression involves more than two types of nominal response variables, and is composed of multiple formulas. For (*j* + 1) types of response variables, there will be *j* formulas, each of which is a binary logit regression process compared with the control group. In addition, in the multiple logit regression, the above *j* binary logit regressions are estimated simultaneously. If the last group is set as the control group and the other groups as the experimental group, *P* (*y_i_* = *j*) is the probability of the experimental group, and *P* (*y_i_* = 0) is the probability of the control group, *β* represents the log odds of the experimental group to the control group, the multivariate logit model of the study can be expressed as:

$$P\left(y_{i}=j\right)=\frac{e^{x_{i}\beta_{j}}}{1+\sum_{k=1}^{j}e^{x_{i}\beta_{k}}},\;P\left(y_{i}=0\right)=\frac{1}{1+\sum_{k=1}^{j}e^{x_{i}\beta_{k}}}$$

In estimating each model, the coefficients of the reference group were normalized to zero. This is because the sum of the probabilities of all choices must be uniform [47,48]. Based on the multivariate logit model, the following probability-relative reference groups can be obtained from the following equation:

$$\frac{P\left(y_{i}=j\right)}{P\left(y_{i}=0\right)}=e^{x_{i}\beta_{j}},\;ln\frac{P\left(y_{i}=j\right)}{P\left(y_{i}=0\right)}=x_{i}\beta_{j}$$

There are also corresponding calculation methods in the parameter estimation process of the unordered multivariate logit selection model. For resident *i*, if route *j* is selected, then *d_ij_* = 1; otherwise, *d_ij_* = 0. At the same time, for resident *i*, only one route can be selected; that is, *d_ij_* = 1. Thus, *y_ij_* (*i* = 1, 2, …, *n*; *j* = 0, 1, 2, …, *m*) and the likelihood function can be obtained. The final log-likelihood function is expressed as follows:

$$ln\;L=\overset{n}{\underset{i=1}{\sum }}\overset{m}{\underset{j=1}{\sum }}d_{ij}\;ln\;P\left(y_{i}=j\right)$$

Based on this, we analyze the preference characteristics of urban forest parks under different location conditions and individual characteristics.

## 3. Results

### 3.1. Participant Background

Among the participants that took part in the survey, the proportion of females was slightly higher than that of males. About half of the respondents were between 20 and 40 years old and the residents sampled in the park were relatively older. More than 80% of the respondents had high school or higher educational backgrounds. More than half of the respondents lived within the Third Ring Road, which was consistent with the expected number and population density of residents residing in the core urban area of Beijing. The total family size of the respondents was three or fewer. The frequency of the respondents visiting the urban forest park was 6–10 times per week, which was related to place of residence (Table 2).

### 3.2. Analysis of Cognitive Map Types

**Figure 4:**
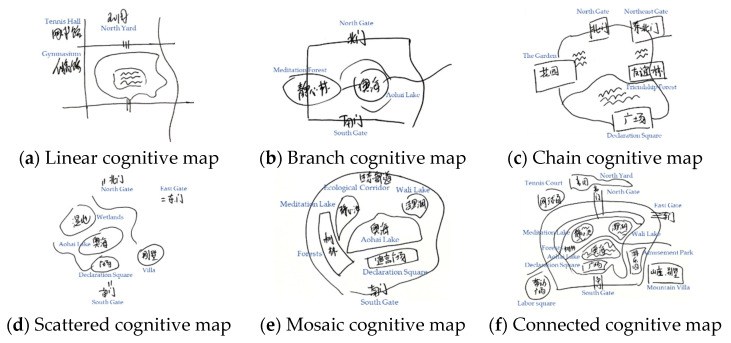
Examples of hand-drawn cognitive maps from this study.

The cognitive maps represent the residents’ preference for the spatial image structure of the entire urban forest park. Based on the cognitive maps, an urban forest park image map was constructed through map type analysis and map element analysis, which were performed to intuitively understand the image composition of urban forest parks. This study used the cognitive map method to analyze residents’ familiarity with the spatial images of different urban forest park routes. Based on the classification criteria proposed by Appleyard (1970), the cognitive maps were roughly divided into sequential cognitive maps, including path elements (such as linear, branch, and chain cognitive maps), and spatial cognitive maps, including regional elements (such as scattered, mosaic, and connected cognitive maps). Examples of hand-drawn cognitive maps in this study are shown in Figure 4.

A linear cognitive map represented a branch map that included the main roads within and surrounding the urban forest park or a more detailed linear preference map. A branch cognitive map was a branch map that contained the elements of the urban forest park which were considered impressive by the interviewees and they could connect the elements through a path. A chain cognitive map was developed based on a chain map around the main roads of the urban forest park. A scattered cognitive map contained a combination of scenic spots or areas with specific impressions, which roughly reflected the preference for the spatial image of an urban forest park. A mosaic cognitive map included different areas of the urban forest park from the respondents’ memory that had to be combined into the preference for the spatial image of an urban forest park. Connected cognitive maps included the spatial segments of urban forest parks connected by the traffic roads and facilities [49].

In this survey, 309 effective cognitive map sketches were obtained. However, only a few samples had good accuracy, while most of them do not form an overall impression of the Olympic Forest Park. When asked to complete the hand-drawn cognitive map, they all said that it was rather difficult. Meanwhile, most of the hand-drawn sketches had incomplete contents, blank areas and negligible additional information, indicating that most of the respondents had no clear understanding of the spatial structure of the Olympic Forest Park. The statistical results obtained from the cognitive maps are presented in Table 3. The specific screening work is the result of cross judgment by experienced investigators and scholars of the research group. Based on the analysis, it was found that 213 were sequential cognitive maps and 96 were spatial cognitive maps. This may be attributed to the differences in the familiarity of the urban forest park areas by residents and their choice of route selection. As residents who exercise in the Olympic Forest Park often chose a specific route as their movement track, their cognition of the park was also potentially affected by their route choices. Therefore, the majority of the cognitive maps obtained were sequential cognitive maps.

Among the sequential cognitive maps, linear cognitive maps account for the largest proportion of 34.30% of the total sample, indicating that most of the residents could draw the main routes and some of the spatial elements and impressive places. Some residents could draw a more detailed linear cognitive map, which was closely related to the residents’ familiarity with the route. Branch cognitive maps obtained from the survey accounted for 20.39% of the total sample. Such maps generally reflected residents’ deep memories of specific places inside and outside the park. Certain scenic spots left a deep impression on residents, who were able to connect these places through various channels and lines to form a roughly branched cognitive map. Chain cognitive maps accounted for 14.24% of the total sample. Some residents could extract chain memory fragments of multiple roads or traffic routes and connect these fragments into a chain-perceptual space.

The scattered cognitive maps accounted for 10.03% of the total sample. This type of map represented several memorable places that roughly reflected the residents’ preference for a certain space. In this case, residents could draw a certain regional boundary of the urban forest park and were able to mark the distribution of multiple important sites. In such maps, residents’ preference for the park’s external or internal element boundary was weak, which was reflected in the presence of scattered elements on the hand-drawn maps. At the same time, residents were able to draw familiar spaces, although there was no clear representation of the overall scope and structure of the park.

In contrast, the mosaic cognitive maps contained more information and was drawn by the residents who are familiar with the park and its surroundings, which accounted for approximately 11.97% of the surveyed park users. Residents integrated memories of different regions into blocks and combined them into the perceived cognitive space. Finally, the connected cognitive map belonged to a higher level, accounting for only 9.06% of the total sample. In this case, residents provided effective connections between segments through necessary traffic lanes and spatial segments to form their perceptual space. According to the interview results, residents who often exercise in the park and live in the surrounding areas could draw various cognitive maps and more mosaic, connection and other complex cognitive maps. In contrast, residents who were not familiar with the park could only draw linear and scattered maps.

### 3.3. Analysis of Cognitive Map Elements

The key elements of cognitive maps can be classified based on classification method proposed by Lynch (1960). In this method, maps are primarily and intuitively assessed based on the richness of certain elements, including landmarks, paths, nodes, regions, and boundaries. The cognitive map elements that residents can feed back are often the most familiar or frequently used parts. By extracting and integrating these elements, we can get the important information related to preferences fed back by residents on the basis of cognitive map. 

Specifically, landmarks in this study were reference points, including famous scenic spots and other elements that could potentially make an impression on residents. Landmarks which emphasize uniqueness are usually spots with specific and easily attractive objects, such as scenic spots, sculptures, buildings or rockeries. Some landmarks exist as symbols in urban forest parks, such as the open-air theater in the Olympic Forest Park. The road within the urban forest park connected different scenic spots or locations, such as greenways, footpaths and ropeways. Roads are an important part of the unconscious or conscious walking route of memory. Hence, it was the dominant element in the cognitive map for most people.

A specific road can serve as an important image feature of parks. A node is a location where residents enter, which connects places, such as squares and parking lots. Nodes may also be the connection points in traffic lines, which play the role of aggregation and transformation. For example, in intersections, squares, bus stations and subway stations, residents can perceive the surrounding environment and make active choices at these nodes. In this study, memories of residents relating to nodes were vague and one of the reasons was the difficulty in remembering names. For example, there are several squares in the Olympic Forest Park, most of which are not well known by residents. Therefore, these squares should be named in a manner that is easily understood and recalled. 

A region is a morphological element on a two-dimensional plane. It divides space with common characteristics into a category. These features can be recognized internally and serve as external references. Boundaries are areas or locations between two regions, such as entrances and exits. Boundaries are often represented as linear elements that divide different areas. Most respondents in this study had vague memories of boundaries, indicating that the residents’ understanding of the Olympic Forest Park was not clear. Thus, it is necessary to consider boundaries while developing urban parks.

**Figure 5:**
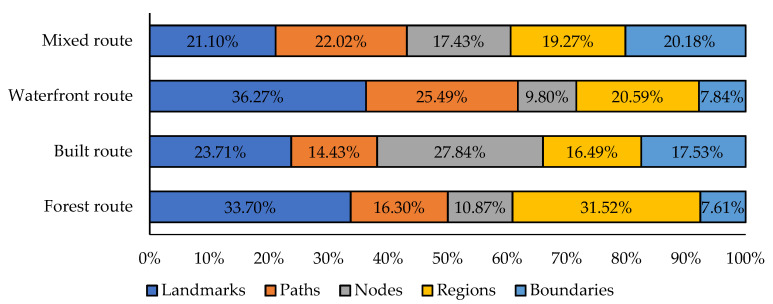
Frequency distribution of familiar units in the cognition maps of four urban forest park routes.

The analyzed information was used to generate the frequency distribution statistics of the cognitive maps of residents based on familiar factors. Figure 5 shows the distribution of the familiarity factors in the cognitive maps. Overall, residents were more familiar with the interior landmarks and regional elements of forest parks, the nodes and boundaries of suburban parks, as well as the external landmarks and paths of urban parks. 

**Figure 6:**
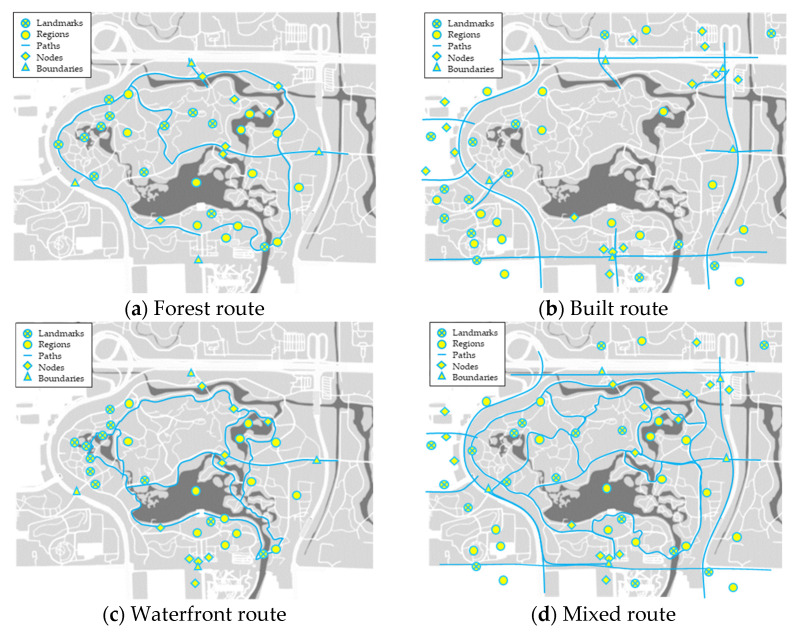
Examples of familiar units in the cognition maps along the four urban forest park routes.

This study evaluated the familiarity of residents with spatial image preference by integrating familiar elements in the cognitive maps of four urban forest park routes. By classifying the five dimensions of cognitive map image elements (i.e., landmarks, paths, nodes, regions and boundaries), the most prominent elements of the urban forest park were identified (Figure 6). In general, residents’ cognitive maps reflect their understanding of the main characteristics of the park and their scope of activities. Apart from residents who preferred the mixed routes, most residents tended to shift their daily exercise route, and only few people shifted their regular route to carry out other activities. Based on the comparison with Figure 2, the information fed back by familiar units in the cognition maps along the four urban forest park routes is consistent with the characteristics of the corresponding urban forest park routes. This behavior of residents may inspire future development of urban forest parks. For example, the tour routes, business arrangements and public service facilities of the Olympic Forest Park can be adjusted and optimized based on residents’ preference and can be combined with their individual characteristics.

1. The familiar units of residents who prefer the forest routes are mainly the landmarks and regions along the route, but most of them are scenic spots and various exercise places in the park, while residents usually pay less attention to the relevant contents outside the park. Generally long and simple, paths in the forest route are internal routes, which are suitable for residents preferring to exercise for long distances. Residents utilizing this route clearly understand the main entrance and exit nodes, but it is not comprehensive enough, which has a certain relationship with a resident’s working and residential location, living habits and exercise habits. According to the interview, the forest route is not only a traditional exercise route based on the resident’s impression, but also an inherent route choice. For such routes, elaborate development strategies should be adopted, which is related to the demand for continuous optimization of park functions, on the one hand, and to make the park more suitable for the core needs of residents, on the other hand.
2. Residents who prefer the built route pay greater attention to the landmarks, regions and nodes outside the park, including nearby gymnasiums, shopping malls, schools and parking lots. This may be because residents have less contact with the scenic spots inside the park except for the entrance and exit areas of the park since the built routes are mostly outside the park fence. Hence, residents have a very clear understanding of boundaries, such as park entrances and exits. Meanwhile, residents are not familiar with paths inside the park but the municipal roads outside the park, such as the North Fifth Ring Road. They have relatively weak demand for the internal functions of urban forest parks. Their demands are mostly outside the park boundary and more diversified. The enlightenment from these residents is that the construction of urban forest park needs to take into account both the internal and external development so as to ensure that the residents’ all kinds of use will not be affected.
3. Residents who prefer the waterfront route have a small range of activities centered on various lakes in the park. These residents are very acquainted with the landmarks and regions around the lake and they are more familiar than those who prefer forest routes. The action tracks of these residents are relatively single and their cognition of boundaries and nodes is mostly concentrated near the park’s south gate. Most of these residents go in and out of the park’s south gate because the largest lake (the Aohai Lake) in the Olympic Forest Park is very close to the south gate. In terms of paths, the residents who prefer the waterfront route pay more attention to the paths inside the park and could provide more details of these paths because the distribution of lakes in the park are relatively scattered, which is similar to the residents who prefer the forest route. At the same time, many river courses are in the same direction as the movement route of residents, which also deepens residents’ familiarity with such water areas. Considering the complexity of the geographical characteristics of the lake and the irregularity of the route around the lake, it is difficult to manage this route. We believe that some improvement measures should be taken from the perspective of strengthening safety guidance and highlighting the landscape characteristics along the route.
4. Residents who prefer mixed routes have a comprehensive understanding of the internal and external elements of the park, but their cognition which is a combination of the above three types of routes is scattered. The movement tracks of these residents are relatively random, and their needs in the park are rather diverse. Therefore, these residents do not have a fixed and habitual exercise route. Conversely, it was understood from the interview that the urban forest park itself has a vivid landscape, but its internal road system was often chaotic, which is the reason why some residents preferred mixed routes. The mixed routes given in this study are also the most used in real life and do not represent all of these routes. When such routes are managed and maintained, it is necessary to fully consider the functions of the other three routes so as to achieve the coordination between their different characteristics.

### 3.4. Effects of Factors on Preferences

Model fitting and uncorrelation tests were performed for established multiple logit models. The results of both tests are shown in Table 4. In the model fitting test, the significance of all explanatory variables of the model was less than or equal to 0.1 or 0.05, indicating that most explanatory variables have an important impact on the equation and the assumption that their variable coefficients are zero can thus be rejected. At the same time, the likelihood ratio index value was significant at the level of 0.01, indicating that the constructed econometric model has a good explanatory effect. The McFadden decision coefficient was 0.152, suggesting that the alternatives meet the irrelevant independence assumption, which means the utility of each route is independent and will not affect each other. In conclusion, the alternatives of this study can meet the requirements of parameter estimation using a logit model.

Based on the conclusions of the fitting and uncorrelation tests, this study used the maximum likelihood method to estimate the parameters of the influencing factors of the preferences of residents. Y1 (Forest route), Y2 (Built route) and Y3 (Waterfront route) were the experimental group, and Y4 (Mixed route) was the reference group. All explanatory variables were included in the model calculation, and then the variables with zero parameter estimation in each model were eliminated by the Wald test based on the reverse elimination method. The results of the maximum likelihood estimation are listed in Table 5.

This research showed that female residents prefer mixed routes, indicating that women’s demand for urban forest parks is more diversified, and their choice of exercise routes are more random than men’s. In terms of age, residents under the age of 20 and over 40 have a stronger preference for mixed routes. This can probably be attributed to more leisure time and more comprehensive needs for urban forest parks of residents in these age groups. Similarly, families with two people prefer the waterfront route, while families with three tend to choose the mixed route. In addition, residents who visit less than five times have a stronger preference for forest routes, while dwellers who visit more than six times have a stronger preference for mixed routes. What account for may be that the residents who visit the park less often prefer the natural functions of the park. In comparison, the demands of residents who visit the park more often will be more diversified. Hence, their route selection will also show the characteristics of diversity and variability.

## 4. Discussion

This study investigated four types of urban forest park routes to reveal the trade-off between residents’ preferences of natural and artificial exercise routes in urban forest parks and explored their needs for these two types of areas. These routes were based on actual usage of urban forest parks by residents and have strong practical significance. In general, residents preferred forest and built routes than waterfront and mixed routes for actual physical exercising. This showed that the traditional exercise route (i.e., the forest route) was still the main choice for the residents. Some studies have pointed out that preference may be affected by media and education, which are potential means that can influence public preference through awareness and ecological education [50,51,52,53]. When it comes to the design of future urban forest park, it is necessary to consider the protection of ecological environment, landscape sustainability, and practicability. Simultaneously, residents’ understanding must be enhanced to diversify their cognition of urban forest parks and expand their choice range.

Judging from the accuracy of hand-drawn sketch elements, most people did not form an overall impression of the Olympic Forest Park so it was difficult for most of them to draw the plan view of the park. Most of the hand-drawn sketches had incomplete contents, blank areas and less information, as residents did not have a clear understanding of the spatial structure of the park. At the same time, we found that the residents’ preference of the spatial images of urban forest parks varied from individual to individual. However, residents who exercise in the urban forest park usually chose a specific route for motion tracking; the preferred route selection might have affected their cognition of the park, which was reflected in the cognitive maps as most of them were sequential cognitive maps. The linear and branch cognitive maps accounted for 34.30% and 20.39% of the total sample, respectively. In these two types of cognitive maps, most residents could only draw the main routes of their actions, indicating that residents have adequate memories of some places inside and outside the park and could illustrate them as simple drawings. During the survey process, some residents with a vague preference for the spatial image of the urban forest park were supplemented with information regarding familiar elements apart from the hand-drawn map, which greatly expanded the sample size and improved the overall efficiency of the survey.

In addition, the cognitive maps drawn by residents reflected their scope of action and their understanding of the main characteristics of urban forest parks. Residents often prefer their daily exercise routes and their preference for different urban forest park routes will be affected by their exercise habits, environmental preferences, residential location and other factors. Based on the different behavior patterns of residents, there is still scope for adjustment and optimization of the tour routes, commercial arrangements and public service facilities of the Olympic Forest Park. Specifically, the family units of residents who prefer forest routes are mainly the landmarks and regions along the route, but most of them are scenic spots and various exercise places in the park, and residents pay less attention to the relevant contents outside the park. This part has the largest number of residents, indicating that the current urban residents’ cognition of the function of urban forest parks remains the most basic aspect of providing natural and ecological products. Their understanding of the social service function of urban forest parks is not deep enough.

In contrast, residents who prefer the built route pay more attention to landmarks, regions and nodes outside the urban forest park, including nearby gymnasiums, shopping malls, schools and parking lots. These residents have a more superficial understanding of the internal elements and functions of the park since their exercise routes are outside the scope of the urban forest park. The route of these residents is highly replaceable and their dependence on the internal elements of the park is low. In addition, the residents who prefer the waterfront route have a small range of activities and are quite familiar with the landmarks and regions around the lake. The number of such residents is small, and they represent a relatively small number of exercise habits and route selection preference. Residents who prefer mixed routes have a comprehensive understanding of the park’s internal and external elements, but they lack an in-depth understanding and their cognition that integrates the above three types is scattered. Notably, there is a certain correlation between preference of residents and their individual characteristics, such as gender, age, family size, visit frequency and other factors that affect their preference for urban forest park routes. In general, the conclusions given by the multinomial logit model are relatively limited, reflecting the relationship between residents’ individual characteristics and the waterfront route and mixed route.

## 5. Conclusions

With the continuous development of China’s urbanization and the rising demands of urban residents for the ecological environment, increasing attention is being paid to the development of livable cities and towns by urban planners, policymakers, scholars and the public. From the perspective of public cognition and choice, this study examined the Beijing Olympic Forest Park as the research area and obtained data through questionnaires and interviews with residents. This study integrated cognitive map analysis and multivariate logit model to study urban forest parks spatial image preference and preference characteristics under different location conditions and individual characteristics. The results suggest that forest routes are still the primary choice for urban residents. Although familiarity with the spatial image preference for urban forest parks varied from person to person, residents’ choice of route shows certain commonalities, which was reflected in the sequential cognitive maps obtained from them. In addition, residents’ route preference is influenced by their exercise habits, environmental preference and residential location. There is also a certain correlation between residents’ preference and their characteristics. This study provides additional information for planners, developers, engineers, architects and foresters in building a more suitable environment that is aesthetically appealing and ecologically sound for physical exercising. Future research should adopt more combinations of methods to explore deep-seated preference of residents, expand the sample size and scope of the survey and conduct repeat and follow-up experiments when conditions permit to verify and expand the conclusions of relevant research [54,55].

## Figures and Tables

**Table 1:** Variables and rating scores of this study.

Variable	Rating Scores
Y	1 = Y1 (Forest route), 2 = Y2 (Built route), 3 = Y3 (Waterfront route), 4 = Y4 (Mixed route)
Gender	1 = Female, 2 = Male
Age	1 = <20 years old, 2 = 21–40, 3 = 41–60 years old, 4 = >60 years old
Education level	1 = ≤Elementary school, 2 = High school, 3 = College/graduate degree
Location of residence	1 = Within 2nd Ring Rd, 2 = 2nd–3rd Ring Road, 3 = 3rd–4th Ring Road, 4 = 4th–5th Ring Road, 5 = 5th–6th Ring Road
Family size	1 = 1 person, 2 = 2 person, 3 = 3 person, 4 = Above 4 persons
Frequency of visits	1 = 1 time or less per week, 2 = 2–5 times per week, 3 = 6–10 times per week, 4 = Above 10 times per week

**Table 2:** Descriptive statistics of samples (%).

Variable	Frequency (%) (N = 803)	Variable	Frequency (%) (N = 803)
Y1 (Forest route)	290 (34.56%)	Location of residence	
Y2 (Built route)	216 (25.74%)	Within 2nd Ring Rd	333 (39.69%)
Y3 (Waterfront route)	150 (17.88%)	2nd–3rd Ring Road	203 (24.2%)
Y4 (Mixed route)	183 (21.81%)	3rd–4th Ring Road	173 (20.62%)
Gender		4th–5th Ring Road	83 (9.89%)
Female	421 (50.18%)	5th–6th Ring Road	47 (5.6%)
Male	418 (49.82%)	Family size	
Age		1 person	59 (7.03%)
<20 years old	75 (8.94%)	2 persons	364 (43.38%)
21–40 years old	407 (48.51%)	3 persons	231 (27.53%)
41–60 years old	226 (26.94%)	Above 4 persons	185 (22.05%)
>60 years old	131 (15.61%)	Frequency of visits	
Education level		1 time or less per week	138 (16.45%)
≤Elementary school	17 (2.03%)	2–5 times per week	167 (19.9%)
High school	680 (81.05%)	6–10 times per week	350 (41.72%)
College/graduate degree	142 (16.92%)	Above 10 times per week	184 (21.93%)

**Table 3:** Statistics of the cognitive maps.

	Map Types	Sequential Cognitive Maps			Spatial Cognitive Maps		
Route Types		Linear	Branch	Chain	Scattered	Mosaic	Connected
1. Forest route		36 (46.15%)	16 (20.51%)	9 (11.54%)	6 (7.69%)	7 (8.97%)	4 (5.13%)
2. Built route		34 (43.59%)	18 (23.08%)	12 (15.38%)	3 (3.85%)	9 (11.54%)	2 (2.56%)
3. Waterfront route		20 (25.64%)	13 (16.67%)	14 (17.95%)	10 (12.82%)	11 (14.10%)	10 (12.82%)
4. Mixed route		16 (20.51%)	16 (20.51%)	11 (14.10%)	12 (15.38%)	10 (12.82%)	13 (16.67%)
5. Average		34.30%	20.39%	14.24%	10.03%	11.97%	9.06%

**Table 4:** Results of the model-fitting test and the independent test.

Variable	Model Fitting Standard	Likelihood Ratio Test		
	The −2ln Likelihood Value of the Simplified Model	Chi-Square Value	df	Significant Level
C	1490.568	0.000	0	-
Gender	1501.253	10.684	3	**
Age	1514.964	24.396	9	***
Education level	1551.558	60.990	6	***
Location of residence	1556.518	65.950	12	***
Family size	1567.104	76.536	9	***
Frequency of visits	1557.818	67.250	9	***
Likelihood ratio		346.849 ***		
McFadden coefficient		0.152		

Note: ** represents the 95% significance level, and *** represents the 99% significance level.

**Table 5:** Maximum likelihood estimation of multinomial logit regression for four urban forest park routes.

Variable	Multinomial Logit Model (N = 803)									Choice of Routes
	$\ln\frac{P\left(y_{i}=1\right)}{P\left(y_{i}=4\right)}$			$\ln\frac{P\left(y_{i}=2\right)}{P\left(y_{i}=4\right)}$			$\ln\frac{P\left(y_{i}=3\right)}{P\left(y_{i}=4\right)}$			
	B	Wald	Exp(B)	B	Wald	Exp(B)	B	Wald	Exp(B)	
C	−1.065	3.521	-	−0.233	0.180	-	−36.877	0.000	-	-
Female	−0.571 ***	6.666	0.565	−0.715 ***	9.885	0.489	−0.856 ***	8.694	0.425	(4)
Age: <20 years old	−0.758 **	3.063	0.469	−0.238	0.305	0.788	−0.235	0.201	0.791	(4)
Age: 21–40 years old	0.516 *	2.709	1.675	0.479	2.233	1.614	0.689 *	2.900	1.992	(3)
Age: 41–60 years old	−0.257	0.614	0.774	−0.207	0.380	0.813	−0.762 *	2.730	0.467	(4)
Family size: 2 people	−0.581 **	3.907	0.559	−0.031	0.011	0.969	0.566	1.978	1.761	(3)
Family size: 3 people	−0.690 **	5.187	0.502	−0.630 **	3.951	0.533	−0.377	0.719	0.686	(4)
Frequency of visits: 1 time or less per week	1.076 ***	7.261	2.934	0.581	2.090	1.787	0.982 **	4.500	2.670	(1)
Frequency of visits: 2–5 times per week	1.618 ***	20.480	5.044	0.211	0.306	1.234	1.138 **	6.461	3.121	(1)
Frequency of visits: 6–10 times per week	−0.026	0.009	0.974	−0.350	1.666	0.705	−0.875 **	5.695	0.417	(4)

Note: * represents the 90% significance level, ** represents the 95% significance level, and *** represents the 99% significance level. Routes (1) to (4) represent the forest, built, waterfront and mixed routes.

## Data Availability

The original data used in this study comes from the empirical research of the research group. The relevant empirical research is funded by Economic Development Research Center of State Forestry and Grassland Administration, Shanghai Normal University and Beijing Philosophy and Social Science Planning Office, which is described in detail in the Funding section. The research group has the right to use the data.
